# Real-Time Digital Signal Processing Based on FPGAs for Electronic Skin Implementation †

**DOI:** 10.3390/s17030558

**Published:** 2017-03-10

**Authors:** Ali Ibrahim, Paolo Gastaldo, Hussein Chible, Maurizio Valle

**Affiliations:** 1 Department of Electrical, Electronic and Telecommunication Engineering and Naval architecture (DITEN)-University of Genoa, via Opera Pia 11, 16145 Genoa, Italy; Paolo.gastaldo@unige.it (P.G.); Maurizio.valle@unige.it (M.V.); 2 MECRL Lab, PhD School for Sciences and Technology (EDST)-Lebanese University, AL Hadath, Lebanon; hchible@ul.edu.lb

**Keywords:** electronic skin system, digital signal processing, FPGA implementation, real-time classification, power consumption

## Abstract

Enabling touch-sensing capability would help appliances understand interaction behaviors with their surroundings. Many recent studies are focusing on the development of electronic skin because of its necessity in various application domains, namely autonomous artificial intelligence (e.g., robots), biomedical instrumentation, and replacement prosthetic devices. An essential task of the electronic skin system is to locally process the tactile data and send structured information either to mimic human skin or to respond to the application demands. The electronic skin must be fabricated together with an embedded electronic system which has the role of acquiring the tactile data, processing, and extracting structured information. On the other hand, processing tactile data requires efficient methods to extract meaningful information from raw sensor data. Machine learning represents an effective method for data analysis in many domains: it has recently demonstrated its effectiveness in processing tactile sensor data. In this framework, this paper presents the implementation of digital signal processing based on FPGAs for tactile data processing. It provides the implementation of a tensorial kernel function for a machine learning approach. Implementation results are assessed by highlighting the FPGA resource utilization and power consumption. Results demonstrate the feasibility of the proposed implementation when real-time classification of input touch modalities are targeted.

## 1. Introduction

The skin is one of the main organs of the human body; it helps us to interact with our surroundings through implementing many different and relevant functions, e.g., protection of the inner body organs, detection of cutaneous stimuli, etc. The skin represents the human physical barrier, allowing us to perceive various shapes and textures, changes in temperature, and varying degrees of contact pressure. To achieve high sensing capabilities, several different types of highly-specialized sense receptors are embedded within our skin. These receptors first transduce information generated by mechanical stimuli into electrical signals and then transmit them to the central nervous systems for more complex processing. The collected signals are eventually interpreted by the somatosensory cortex, [1] permitting us to perceive the sense of touch and to easily interact with our physical world. 

The development of electronic skin (e-skin) is a very complex and challenging goal which involves many different and complementary research areas. Nonetheless, the effort to create an e-skin with human-like sensory capabilities is motivated by the possibility of being highly applicable for autonomous artificial intelligence (e.g., robots), biomedical instrumentation, and replacement prosthetic devices capable of providing the same level of sensory perception of the organic equivalent. 

**Figure 1:**
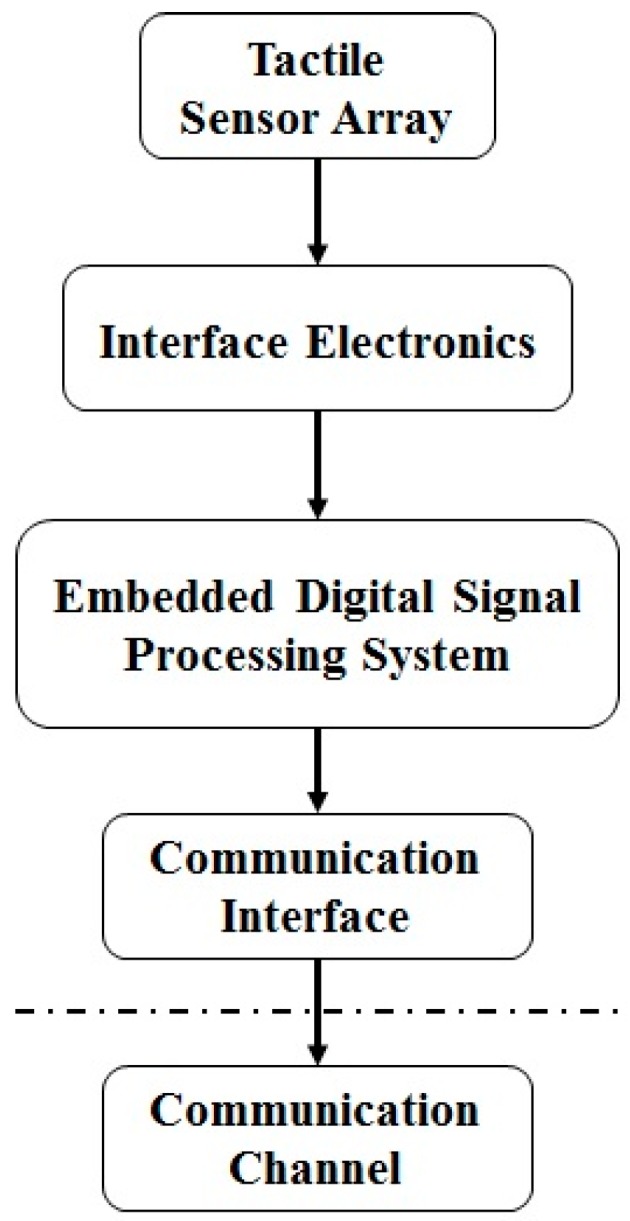
Structural block diagram of an e-skin system.

Following the definition given by Dahiya et al. [2], tactile sensing involves the detection and measurement of contact parameters in a predetermined contact area and subsequent processing of the signals to extract structured and meaningful information which is subsequently transmitted to higher system levels for perceptual interpretation. Figure 1 provides a structural block diagram of an e-skin system. The development of the e-skin system starts by defining the system specifications, designing and fabricating the mechanical arrangement of the skin itself (i.e., sensing materials), together with the embedded digital system for tactile data processing. The different e-skin tasks are still in their infancy and far from being properly addressed even if many research groups are addressing the topic with different approaches at each level of the problem [3,4,5,6,7,8,9].

Significant progress in the development of e-skin has been achieved in recent years by the concentration on mimicking the mechanically compliant highly sensitive properties of human skin. For the sensing materials, stretchable electrodes for e-skin have been developed in [10], and the transformation of a typically brittle material, Si, into flexible, high-performance electronics by using ultrathin (100 nm) films connected by stretchable interconnects is presented in [11]. Someya et al. have fabricated flexible pentacene-based organic field-effect transistors (OFETs) for large-area integrated pressure-sensitive sheets with an active matrix readout [12]. For the system implementation, however, the design of a tactile sensor patch to cover large areas of robots and machines that interact with human beings is reported in [13]. The realizations are mostly custom-built and the sensor is implemented with commercial force sensors. This has the benefit of a more foreseeable response of the sensor if its behavior is understood as the aggregation of readings from all of the individual force sensors in the array. Mittendorfer et al. [14] introduced a cheap, scalable, discrete force cell and integrated it, along with other (discrete) sensor devices, into a multi-modal artificial skin, based on hexagonal-shaped, intelligent unit cells (i.e., PCBs). However, the very large amount of data, the complexity of data processing algorithms, and the relevant amount of energy and area restrict the current implementations of e-skin systems to networked PCB systems. 

This paper presents the architecture of an electronic skin system designed to be hosted on embedded devices. The overall system includes both the e-skin layer and the underlying pattern-recognition module, which is entitled to support smart tactile-sensing functions. In the proposed system, pattern recognition is implemented by exploiting machine-learning (ML) methodologies, which have already been proved able to tackle complex touch-recognition tasks [15,16,17,18,19,20]. In particular, the present pattern-recognition device takes advantage of an approach that can deal with the inherent tensor morphology of raw tactile data [18]. The proposed architecture demonstrates the feasibility of the approach despite the hardware complexity when real-time functionality is aimed. Moreover, the paper highlights the high amount of power consumption needed for the input touch modalities classification task and proposes possible solutions for effective implementation of e-skin systems. 

The rest of the paper is organized as follows: Section 2 describes the e-skin from a systems perspective, defining the different structural components for the system development. Section 3 introduces the pattern recognition model exploiting machine learning methodologies. The section describes a tensor-based framework for tactile data. Section 4 presents the digital signal processing computational architecture for the tensorial approach. It analyzes the computational load of the proposed approach and provides the hardware implementation results based on FPGA device. A classification study based on hardware implementation results is elaborated in Section 5, and finally conclusions and future perspectives are reported in Section 6.

## 2. Electronic Skin: A Systems Perspective 

**Figure 2:**
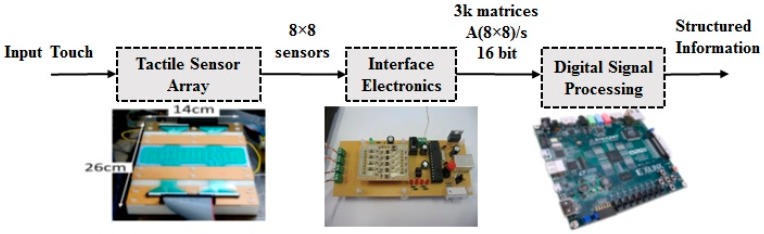
Block diagram of the e-skin system prototype. Courtesy of COSMIC lab at the University of Genova, Italy (http://www.cosmiclab.diten.unige.it/).

From a systems perspective, e-skin is usually defined as a set of multiple sensing components, including structural and functional materials, signal conditioning, and acquisition, integrated with a dedicated sensor information processing embedded electronic system [21]. Figure 2 shows a block diagram of the e-skin system prototype. 

### 2.1. Tactile Sensor Array

A tactile sensor array is the first component to be addressed in the e-skin development process. The adequate functional material enabling certain sensing capabilities should be identified. As the functional skin requirements are debatable and application-dependent, piezoelectric polymer films of polyvinylidene fluoride (PVDF) [22] have been chosen as meeting the target requirements of mechanical flexibility, high sensitivity, detectability of dynamic touch (1 Hz–1 kHz frequency range) and robustness. 

Commercial PVDF sheets (100 um thick) from Measurement Specialties Inc. (Norristown, PA, USA) are stretched and poled. Stretching at temperatures below the polymer melting point and poling by the application of very high electric fields (~100 V/μm) give the polymer sheets the symmetry of an orthotropic material [22]. Linear constitutive equations [22] are commonly used to describe the material’s intrinsic transduction of the mechanical stimulus into a charge signal, but care is required to account for the way the piezoelectric film is integrated into the skin, which also includes a substrate and a cover layer. The system consists of an 8 × 8 tactile sensor array based on piezoelectric transducers [23]. 

### 2.2. Interface Electronics

The second block consists on the interface electronics which is in charge of signal conditioning and acquisition including the analog to digital conversion [24]. The interface electronics requires a charge amplifier to collect the charge generated by the PVDF single taxel when stressed by tactile stimuli. The charge amplifier transfers the charge to a reference capacitor and produces an output voltage which is proportional to the charge on the reference capacitor and, respectively, to the input charge; hence, the circuit acts as a charge-to-voltage converter. The charge amplifier may amplify the tactile stimuli in the frequency band of interest which, for our case, is in the range from 1 Hz to 1 kHz. The analog to digital converter scans the 8 × 8 sensor array at the rate of 2 kHz × (OSR) where 2 kHz is the input signal Nyquist frequency and OSR is the oversampling ratio factor which must be larger than 2 (OSR has been set to 3 in the current setup) [25]. The sample rate at the output of the interface electronics block is, consequently, of 3000 matrices (8 × 8)/s, with a nominal data resolution (set by the analog to digital converter) equal to 16 bits. The relation between the signal to noise ratio (SNR) and the effective number of bits (ENOB) is defined by the following equation: ENOB = (SNR − 1.76)/6.02. The SNR for different values of the contact force at 1 kHz are experimentally reported in [24], the resulting ENOB is equal to 8; the same value has been set for the present setup. Tactile sensors data have to be processed and structured information needs to be extracted and transmitted. 

### 2.3. Digital Signal Processing

The digital signal processing (DSP) role is elaborates the tactile sensor signals using an embedded electronic system integrated together with sensing materials. Tactile data processing concerns different kinds of information which could be divided into two categories: (1) low-level information, such as contact location, area and duration, contact force intensity, direction and distribution, and temperature; (2) high-level information for discrimination of the touch modality or the classification of attributes of the contacting objects, e.g., roughness, textures, patterns, etc. In the present setup, the e-skin system deals with DSP block with high level information processing namely input touch modality classification. The classification uses machine learning based on a tensorial kernel approach, which has recently proven its effectiveness in processing tactile sensor data [26]. 

## 3. The Pattern Recognition Model 

Machine-learning techniques can support the design of predictive systems that make reliable decisions on unseen input samples [27,28]. This ability is attractive whenever the underlying phenomenon to be modelled is complex; i.e., when an explicit formalization of the input-output relationship is difficult to attain. Actually, ML can model the input-output function by a “learning from examples” approach. Eventual implementations can vary according to different application scenarios, but all share a common probabilistic setting. 

In the case of a tactile-sensing framework, the problem is to interpret the sensor signals to discriminate between a set of stimuli that the system is expected to recognize. ML techniques may indeed face challenging assignments such as the discrimination of materials or the interpretation of touch modalities. To this purpose, one can reduce the overall complexity of the pattern recognition problem by splitting the modelling process into two tasks:

1. The definition of a suitable descriptive basis for the input signal provided by the sensor (or lattice of sensors), i.e., a feature-based description that lies in a feature space F:
  (1) ϕ(S)→F
  In Equation (1), ***S*** is the third-order tensor that characterizes sensor outputs.
2. The empirical learning of a model for the non-linear function, *ξ*, that maps the feature space, F, into the set of tactile stimuli of interest:
  (2) ξ:F→T
  In this research, T includes a finite number of stimuli, hence, *ξ* implies a multi-class classification task.

The literature provides a wide range of ML-based techniques to set up *ξ*. On the other hand, the peculiarities of a tactile-sensing framework notably shrink the range of solutions that best fit the underlying three-dimensional tensor problem. In fact, the large majority of ML paradigms is designed to support framework that processes *n*-dimensional vectors that lie in some feature space $F\subset R^{n}$. This, in turn, means that one would need a feature-extraction process that significantly alters the original structure of the signal provided by the sensor, since $S\subset R^{r}⊗R^{c}⊗R^{d}$.

In this paper, the setup of *ξ* is supported by a theoretical approach that can lead to a tensor-oriented kernel machine. That is, the function *ξ* is learned by using a support vector machine (SVM) [27] that can inherently process tensors, rather than *n*-dimensional vectors. Accordingly, the pattern-recognition module can benefit from (1) a powerful machine-learning paradigm (SVM); and (2) a suitable processing of sensors data. 

### 3.1. SVM

The empirical learning of the mapping function *ξ* stems from a training procedure that uses a dataset, **X**, holding *N_p_* patterns (samples). In a binary classification problem, each pattern includes a data vector, x∈R, and its category label *y* ∈ {−1, 1}. When developing data-driven classifiers, the learning phase requires both ***x*** and *y* to build up a decision rule. After training, the system processes data that do not belong to the training set and ascribes each test sample to a predicted category $\hat{y}$. 

According to the SVM model, the function that predicts the class of a sample is a sharp decision function, $\hat{y}=\mathrm{sign}(f(x))$, where *f*(***x***) is a weighted sum of some nonlinear “kernel” basis functions. A kernel function *K*(***x****_i_*, ***x****_j_*) allows to handle only inner products between pattern pairs, disregarding the specific mappings of individual patterns [27]. The kernel trick allows setting up the non-linear variant of virtually any algorithm that can be formalized in terms of dot products. 

Actually, one has:

(3) $$f(x)=\sum_{i}^{Nsv}\alpha_{i}y_{i}K(x_{i},x)+b$$

where the number of support vectors *N_sv_* , the “bias” term *b*, and coefficients α_i_ are computed by the training algorithm [27], which minimizes a quadratic cost function [27]. The eventual generalization performance of a SVM depends also on the specific setting of the scalar parameter *C* that regulates the trade-off between accuracy and complexity in the training process [27].

### 3.2. A Kernel Function for Tensors

The theoretical framework presented in [29] introduced a kernel function for developing tensor-based models. Such a result is noteworthy in that it allows every kernel machine to deal with tensors. This goal is achieved by designing a suitable kernel that can exploit the algebraic structure of tensors. As a major consequence, one can rewrite Equation (3) by making use of a kernel $K(\chi_{i},\chi )$, where $\chi_{i},\chi$ are tensors rather than n-dimensional vectors.

In [29], the kernel function K(A,B) that processes two generic tensors $A,B\in R^{I_{1}\times I_{2}\times \cdot \cdot \cdot \times I_{N}}$ is formulated as:

(4) $$K(A,B)=\prod_{n=1}^{N}K^{\left(n\right)}(A,B),$$

where:

(5) $$K^{\left(n\right)}(A,B)=\exp\left(-\frac{1}{\sigma^{2}}(I_{n}-\mathrm{trace}(Z^{t}Z))\right)$$

(6) $$Z={{\overset{⌢}{V}}_{\left(n\right)}^{A}}^{t}{\overset{⌢}{V}}_{\left(n\right)}^{B}$$

In Equation (6), ${\hat{V}}_{\left(n\right)}^{B}$ is the matrix computed by applying singular value decomposition (SVD) to $B_{\left(n\right)}$, which corresponds to the mode-*n* unfolding of B. First, the SVD remaps the original matrix $B_{\left(n\right)}\in R^{I_{n}\times I_{1}I_{2}\cdot \cdot \cdot I_{n-1}I_{n+1}\cdot \cdot \cdot I_{N}}$ into a coordinate system where the covariance matrix is diagonal. Thus:

(7) $$B_{\left(n\right)}=U_{n}\Sigma_{n}{V_{n}}^{t},$$

where $V_{\left(n\right)}^{t}$ is a (*I*_1_*I*_2_^…^*I*_*N*−1_*I*_*N*+1_^…^*I_N_*) × (*I*_1_*I*_2_^…^*I*_*N*−1_*I*_*N*+1_^…^*I_N_*) orthogonal matrix. Then, ${\hat{V}}_{\left(n\right)}^{B}$ is obtained by selecting the first *r* columns of $V_{\left(n\right)}^{t}$, with *r* = rank($B_{\left(n\right)}$). A similar procedure applies to ${\hat{V}}_{\left(n\right)}^{A}$.

Overall, the computation of K(A,B) requires a set of 2*N* SVD’s (as both A and B are processed). Then, two matrix products for each $K^{\left(n\right)}(A,B)$ must be performed. The implementation of the kernel-based decision function, Equation (4), requires a set of *N_sv_* inner products *K*(∙,∙), involving the test pattern versus all training patterns. On one hand, the SVD result of each training pattern is computed offline and is stored in memory; however, the SVD of the test pattern has to be worked out online. Thus, the computational cost ***O***_PRED_ associated to such step is:

***O**_PRED_* ≅ *N*∙***O**_SVD_* + 2∙*N_sv_*∙*N*∙***O**_MP_* (8)

where ***O**_SVD_* and ***O**_MP_* are the computational costs of a SVD and a matrix product, respectively.

### 3.3. A Tensor-Based Framework for Tactile Data

The paper of Gastaldo et al. [18] showed that a pattern recognition framework based on the tensorial SVM can effectively tackle touch modality classification. The corresponding machine learning system models the mapping function *ξ* by using a dataset holding *Np* patterns (samples), where each pattern now includes a data tensor χ and its category label *y* ∈ {−1, 1}. This allows keeping the original training procedures adopted by the SVM model. As a result, Equations (1) and (2) can be reformulated as follows:

(9) χ=ϕ(S)

(10) ξ:χ→Τ

where χ is a tensor space. The process *φ*(9) now can work out a tensor-based description from ***S***, thus preserving the structure of the signal originally provided by the tactile sensor. In principle, the learning system, Equation (4), could be designed to receive as input the tensor ***S*** directly. In fact, pre-processing may be needed to better characterize the underlying tactile phenomenon. In [18], two different pre-processing approaches have been suggested. 

The experimental results provided in [18] proved that “tensor-SVM” could obtain consistent performances on a three-class classification problem. The experimental evidence seemed to confirm that the availability of a tensorial kernel function may prove valuable when tackling classification problems that admit a natural multiway representation. 

## 4. Digital Signal Processing Implementation

The implementation of real-time embedded electronic system based on the tensorial framework described in Section 3.3 is targeted for the DSP of the electronic skin system.

As shown in the block diagram of the Figure 2, the input of the digital signal processing block is 3000 matrices (8 × 8)/s which represents a data arrangement in terms of a time stream of arrays, i.e., as a third-order tensor £(8 × 8 × 3000), where the first two dimensions are defined by the geometry of the sensor array (8 × 8), while the time defines the third tensor dimension. The high amount of data contained in the tensor £(8 × 8 × 3000) is reduced according to the method proposed in [18] in order to reduce the complexity of computation. Applying this method to the input tensor results a reduced tensor *φ*(8 × 8 × 20). 

**Figure 3:**
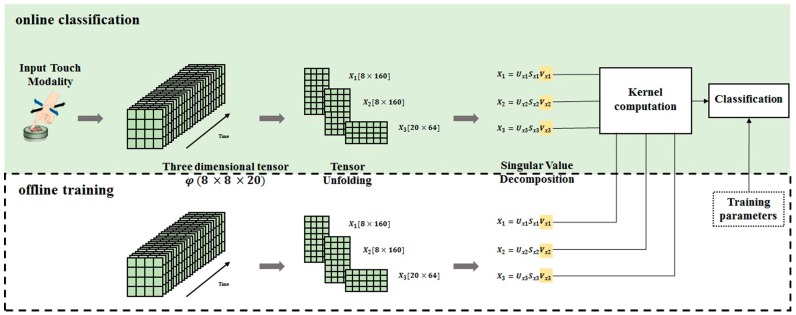
Computation steps for the tensorial kernel approach.

Figure 3 shows a sketch of the different computation steps needed to classify input touch modalities using the tensorial approach. The approach is applied following two phases: offline learning and online classification. During the offline learning, the training is done using a dataset holding a number of samples to build up a decision rule and hence developing data-driven classifier. Thus, in the online classification phase, the system computes the distances between the data from the input touch modality and that do belong to the training set; using the offline developed classifier, the system classifies each input sample with a predicted category. 

**Figure 4:**
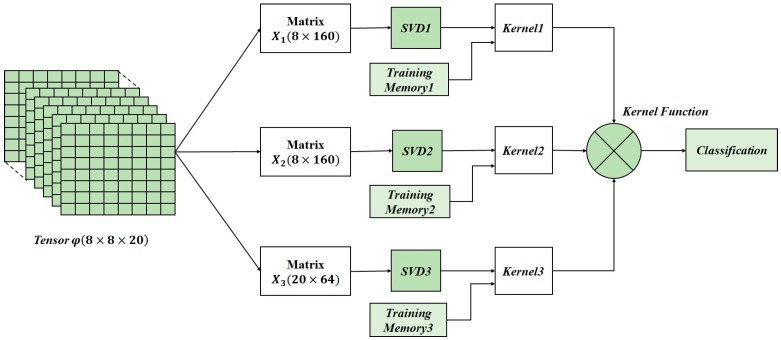
Online computation architecture for the tensorial kernel approach.

The DSP deals with the online classification phase of the approach. The online computation architecture for hardware implementation is described in Figure 4. The first computational step consists on tensor unfolding i.e., a matrix representation of *φ*(8 × 8 × 20) where all the column (row) vectors are stacked one after the other [30]. Three matrices (**X**_1_ (8 × 160), **X**_2_ (8 × 160), **X**_3_ (20 × 64)) are obtained by applying unfolding. Then, the SVD blocks compute the singular value decomposition which transforms the unfolded matrices into the product of three matrices, e.g., **X**_1_ = **U**_1_**S**_1_**V**_1_^T^ where **U**_1_ is an orthogonal matrix containing the eigenvectors of **X**_1_**X**_1_^T^, and **V**_1_ is an orthogonal matrix containing the eigenvectors of **X**_1_^T^**X**_1_. The S_1_ matrix is a diagonal matrix diag(σ_0_,…,σ*_n_*_−1_), where the σ*_i_* are the singular values of **X**_1_ (i.e., the square roots of the eigenvalues), being arranged in descending order. 

The kernel computation comes into effect after the SVD completion. Kernel computation deals with the kernel factor, which is computed by using the singular vectors (*V_i_*) of the input tensor and the singular vectors of the training tensors for the different classes memorized from the offline training phase. After that, the kernel function is obtained by multiplying the resulted kernel factors for the three unfolded matrices. Finally, the classification is done using the online computed kernel function and the offline memorized training parameters. 

### 4.1. Computational Load Analysis

In addition to the very large amount of tactile data to be processed in real-time, the computation complexity poses a tough challenge in the development of the embedded electronic system. Computational requirements depend on the overall number operations (mainly arithmetic) that the tensorial kernel approach must perform and on the real-time operation. 

In order to assess the computational load, a case study [23] has been considered: the given task is to classify a touch interaction among *Nc* = 3 touch modalities (i.e., paintbrush brushing; finger sliding; washer rolling) in 1 s; here *Nc* is the number of classification classes and the number *Nt* of the training data is set to 100. 

As described in the Figure 3 the approach consists first of computing the singular value decomposition (SVD) [31] of the unfolded matrix. The analysis of the computational requirements for the SVD is based on the one-sided Jacobi algorithm which provides high accuracy and convergence in about *K* = 5:10 iterations. Following step is the computation of the kernel factor for a couple of SVDs, the first corresponding to the tensor input and the second to the tensor representing a predefined class extracted from the training data. Table 1 shows the number of operations and flops per second needed to implement the tensorial kernel approach. The power consumption of the resulted total FLOPS number has been estimated according to [32].

Following estimations presented in Table 1, about 31 GFLOPS (giga-floating point operations per second) are needed for real-time single touch classification. These requirements for the data processing unit are very challenging: an appropriate data processing unit need to be carefully selected in order to meet the target requirements. 

Embedded DSP microprocessors for instance, perform their arithmetic operations via software; this can give the flexibility in design, allowing late design changes. For example, let us consider the very well-known ARM Cortex processor family [33]: the Cortex-R7 can achieve 6 GFLOPS, which is lower than the target requirements highlighted by Table 1. Moreover, power consumption is not compatible with the target application requirements. 

A possible approach to tackle this issue could be to design dedicated application specific integrated circuit (ASIC) on a standard cell technology; to this end, our approach is to use the field programmable gate array (FPGA) which represents an efficient solution combining the strengths of hardware and software. Moreover, prototyping ASIC designs in FPGAs is an effective and economical method of verification.

### 4.2. FPGA Implementation Results

The computational load study results the SVD as the most computational expensive algorithm of the tensorial kernel approach: it represents about 70% of the computational complexity of the overall approach [23]. For this reason, methods and architectures for the hardware implementation of the SVD have to be well studied and assessed in order to select an appropriate architecture suitable for the targeted application. In this perspective, three different hardware implementations for the SVD have been presented and assessed in [34], and an implementation suitable for embedded real-time processing has been selected. 

**Figure 5:**
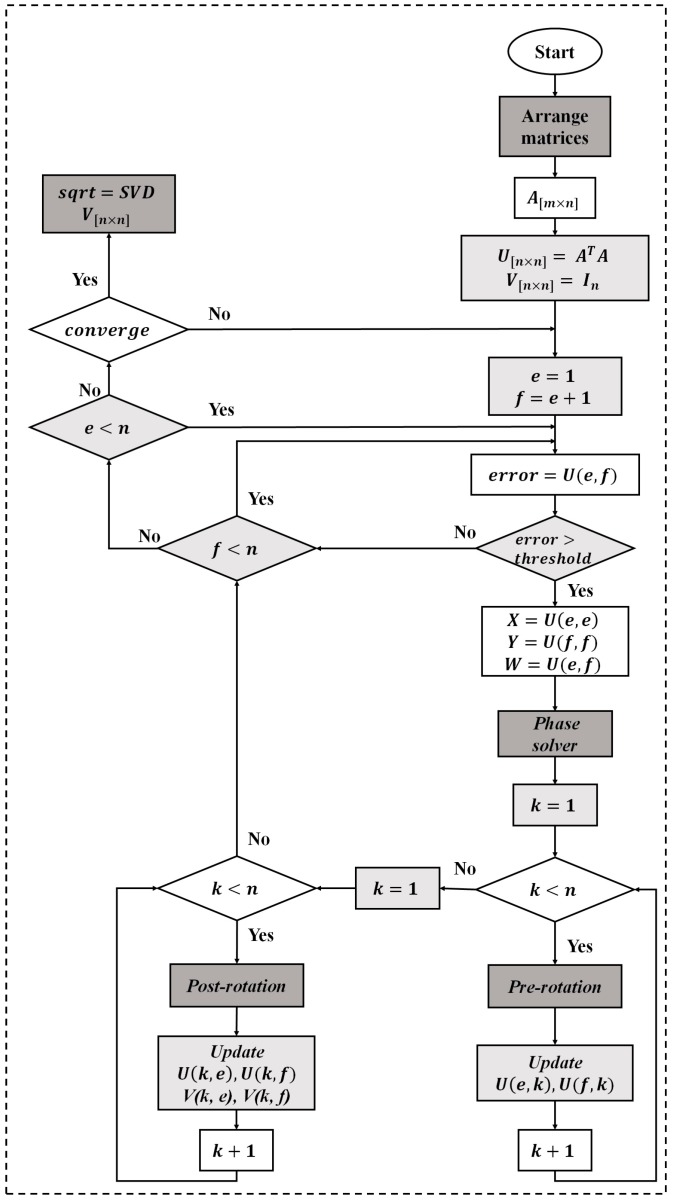
Flow diagram of the singular value decomposition implementation.

The hardware implementation of the SVD is based on the one-sided Jacobi algorithm as shown in Figure 4. The one sided Jacobi algorithm is based on diagonalizing rotations preserving angles and lengths by using orthogonal transformations. The algorithm deals with square and symmetric matrices so a matrix symmetrization scheme should be applied at the beginning of the process as shown in Figure 5. The matrix symmetrization block multiplies the unfolded matrix **X***_i_* by its transpose, resulting a square and symmetrical matrix **U***_n_*_×*n*_ = **X***_i_*^T^**X***_i_*.

The concept of the one-sided Jacobi algorithm is to apply a sequence of rotations to the symmetric matrix **U***_i_*, in order to reach the diagonal matrix **S**. Starting from the *n* × *n* symmetric matrix **U** = **U**_0_, the algorithm produces a sequence **U**_1_, **U**_2_… which eventually converge to a diagonal matrix with the eigenvalues on the diagonal. **U***_i+_*_1_ is obtained each time from **U***_i_* by the transformation given by the formula:

**U***_i_*_+1_ = **J**(*i*, *j*, *θ*)^T^ × **U**_i_ × **J**(*i*, *j*, *θ*) (11)

where **J**(*i*, *j*, *θ*) is called a Jacobi rotation. 

The Jacobi rotation **J**(*i*, *j*, *θ*) is introduced, for an index pair (*i*, *j*) and a rotation angle *θ*, as a square matrix that is equal to the identity matrix I plus four additional entries at the intersections of rows and columns *i* and *j*. **J**(*i*, *j*, *θ*) is calculated on every 2 × 2 matrix to zero out all non-zero off-diagonal elements of the symmetric matrix. 

Cyclic Jacobi method [35] provides an inexpensive computational approach to compute the transformations, it consists in organizing the computations in sweeps within which each matrix element is annihilated once, and each sweep consists of *n*(*n* − 1)/2 transformations. The one-sided Jacobi computes the SVD through a pre- and post-multiplication by the Jacobi rotation matrix. For that, the complexity of this algorithm lies in the computation of the phase solver block and in the management of the rotations represented by pre- and post-rotation blocks of the Figure 5. The SVD hardware implementation results based on Virtex-5 XC5VLX330T FPGA device (Xilinx Inc., San Jose, CA, USA) are shown in Table 2. The architectures and FPGA implementation details of the SVD can be found in [31].

Using the proposed SVD implementation, the computation of the kernel function according to (5) has been pursued. Table 3 shows the implementation results of the kernel function using a Virtex-5 XC5VLX330T FPGA device. The results correspond to one kernel function computed for an input tensor compared with an only one training tensor belongs to one class.

## 5. Classification Study Based on FPGA Implementation

Basing the study on the FPGA implementation results, this section assesses the hardware complexity for the real-time classification of input touch modalities. The number training data for the tensorial kernel approach varies roughly between a minimum of *Nt* = 100 and a maximum of *Nt* = 1000 training tensors [23]. In order to provide a reasonable quantification of the hardware resources and power consumption of the approach, two cases are assessed: (1) Classification of three input touch modalities with a number minimum of training data (*Nc* = 3 and *Nt* = 100) which represents the study case presented in Section 4.1; and (2) the classification of five input touch modalities with an average number of training data (*Nc* = 5 and *Nt* = 500). 

### 5.1. Case 1: Nc = 3 and Nt = 100

Let us define the real-time functionality as a time latency less than 1 s so that the system should figure out one classification per second. This case deals with a total of 300 training tensors, so 300 kernel functions must be computed with a time latency less than 1 s. However, the SVD for the input tensor is computed only for the first kernel function, then memorized and used for the remaining kernel function computations the fact which reduces the time latency of the overall system. 

According to Table 2 and Table 3, these computations are conducted with a time latency equal to 1.59 + (1.59 − 0.42) × 299 = 351 ms < 1 s. Thus, using the computational architecture presented in Figure 4 assure the real-time functionality for three input touch modalities with the minimum number of training tensors. Moreover, hardware complexity and power consumption remain unchanged. Thus, the hardware complexity and the power consumption presented in Table 3 are needed to classify three different input touch modalities with 100 training tensors. 

### 5.2. Case 2: Nc = 5 and Nt = 500

**Figure 6:**
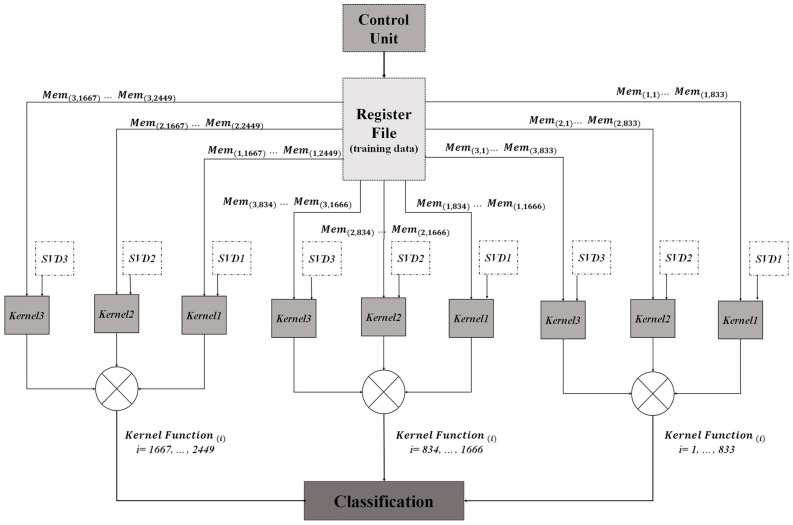
Parallel architecture for the tensorial approach hardware implementation for *Nc* = 5 and *Nt* = 500.

**Figure 7:**
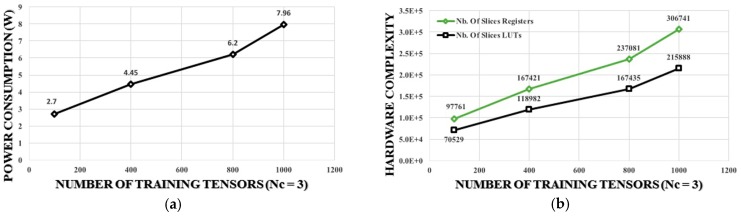
Training tensors versus (**a**) power consumption and (**b**) hardware complexity (*Nc* = 3).

**Figure 8:**
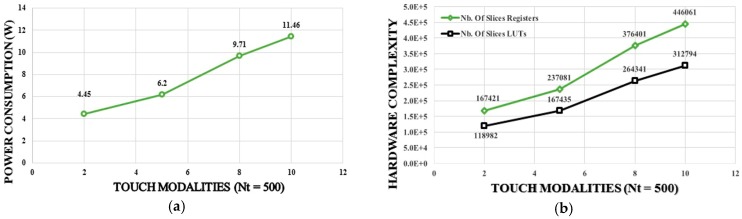
Number of touch modalities versus (**a**) power consumption and (**b**) hardware complexity (*Nt* = 500).

A total of 2500 kernel functions need to be computed in this case. The corresponding time latency is equals to 1.59 + (1.59 − 0.42) × 2499 = 2900 ms > 1 s. Thus, the computational requirements in this case do not satisfy the real-time functionality when using the computational architecture presented in Figure 3. Time latency should be three times reduced to be less than 1 s. This issue can be tackled by implementing a parallel hardware architecture providing three parallelism levels. Figure 6 shows the hardware architecture providing 3 parallelism levels of the computational steps presented by Figure 4. Using this architecture the time latency will be given by 1.59 + (1.59 − 0.42) × 2499/3 = 976 ms < 1 s, and the real-time functionality is confirmed. However, although the proposed parallel architecture assures the real time classification, it increases the hardware resources and the power consumption. Table 4 summarizes the hardware complexity for the real-time classification of the input touch modalities for the two studied cases. The results presented in this table demonstrate the feasibility of the approach for the real time classification. The high amount of power consumption still represents a challenging task to be addressed for the target application since it affects the scalability of the system. Figure 7 and Figure 8 highlight the system scalability in terms of hardware complexity and power consumption. Figure 7 shows the estimation of the variation for the (a) power consumption and (b) the hardware complexity of the proposed implementation in terms of the number of training tensors (*Nt*,) with *Nc* = 3. On the other hand, Figure 8 presents (a) the power consumption and (b) the hardware complexity versus the number of touch modalities (*Nc*) with *Nt* = 500. The figures show a linear relationship between hardware complexity and power consumption and *Nc* and *Nt*. Power consumption may achieve in some cases unacceptable values for a large number of touch modalities, e.g., power consumption of 11.4 W for 10 classes. Hence, more efforts should be dedicated on reducing the hardware complexity and power consumption of the DSP system to deal with embedding constraints. This goal can be pursued by using specific design methods such as approximate computing techniques [36,37,38]. Exploiting inexact arithmetic circuits for SVD implementation would improve the system efficiency by decreasing the power consumption and hardware resources. On the other hand, employing an ASIC implementation using standard cell and a deep submicron technology may considerably reduce the power consumption.

## 6. Conclusions and Future Perspectives

Embedding digital signal processing systems into e-skin for tactile data processing has to comply with severe constraints imposed by the application, e.g., real-time response, low power consumption and small size. In this paper we presented an implementation of DSP-based FPGA for an e-skin system. The DSP deals with machine learning based on a tensorial kernel approach. Implementation results are assessed by highlighting the FPGA resources utilization and power consumption. Results demonstrate the feasibility of the proposed implementation when real-time classification of input touch modalities is targeted. 

When considering the pattern-recognition system, a crucial goal will be the development of a multi-class classification framework that can still reliably address such challenging problem when more touch modalities are involved. On the one hand, the proposed approach can be easily extended to multi-class problems. However, such extension has to be carefully addressed when dealing with the hardware implementation.

The implementation results highlight the high amount of power consumption needed which represent the main issue for the system development. Furthermore, scaling up the system requirements (e.g., the number of classes) may dramatically increase the power consumption; a fact that affects the system efficiency. Hence, the requirements related to the development of embedded data processing unit for e-skin are still far from being achieved with the current methods. Methods and techniques to reduce hardware complexity and power consumption of the embedded DSP system should be investigated. 

A possible solution would be by using approximate computing which has recently emerged as a promising approach to energy efficient design of digital systems [36]. Approximate computing relies on the ability of many systems and applications to tolerate some loss of quality or optimality in the computed result. By relaxing the need for fully precise or completely deterministic operations, approximate computing techniques allow substantially improved energy efficiency.

Another possible solution could be by using many-core architectures such as PULP (parallel processing ultra-low power platform) [39] which have shown promising results on embedded parallel applications, providing state-of-art performance with a reduced power budget. The goal of the PULP platform is to satisfy the computational demands of IoT applications requiring flexible processing of data streams generated by multiple sensors. Such parallel ultra-low-power programmable architecture may allow to meet the computational requirements of the targeted application, without exceeding the power envelope of a few mW typical of miniaturized, battery-powered systems.

## Figures and Tables

**Table 1:** Floating point operations per second (FLOPS).

	Addition/Subtraction	Multiplication	Division	Square Root	Total FLOPS	Power Consumption (W) [32]
Number of operations	$1.56\times 10^{10}$	$1.58\times 10^{10}$	$6.48\times 10^{5}$	$4.32\times 10^{5}$	$3.14\times 10^{10}$	1.04×10

**Table 2:** SVD implementation results for Virtex-5 XC5VLX330T.

Matrix Size	Time Latency (ms)	Percentage Occupied Area (%)	No. of Slice Registers	No. of Slice LUTs	Power Consumption (W)
160 × 8	0.42	18	28,101	22,076	0.948

**Table 3:** Kernel function implementation results for Virtex 5 XC5VLX330T.

Matrix Size	Time Latency (ms)	Percentage Occupied Area (%)	No. of Slice Registers	No. of Slice LUTs	Power Consumption (W)
160 × 8	1.59	74	97,761	70,529	2.709

**Table 4:** Requirements for real-time classification of input touch modalities.

	Time Latency (s)	No. of Slice Registers	No. of Slice LUTs	Power Consumption (W)
*Nc* = 3 and *Nt* = 100	0.35	97,761	70,529	2.7
*Nc* = 5 and *Nt* = 500	0.97	150,604 (estimated)	108,652 (estimated)	6.2
